# Food-Derived Hemorphins Cross Intestinal and Blood–Brain Barriers *In Vitro*

**DOI:** 10.3389/fendo.2018.00159

**Published:** 2018-04-10

**Authors:** Dorothée Domenger, Benoit Cudennec, Mostafa Kouach, Véronique Touche, Christophe Landry, Jean Lesage, Fabien Gosselet, Sophie Lestavel, Jean-François Goossens, Pascal Dhulster, Rozenn Ravallec

**Affiliations:** ^1^Université de Lille INRA, ISA, Université d’Artois, Université Littoral Côte d’Opale, EA 7394-ICV Institut Charles Viollette, Lille, France; ^2^Plateau de Spectrométrie de Masse “PSM-GRITA”, EA 7365, Faculté de Pharmacie, Université de Lille, Lille, France; ^3^Université de Lille INSERM, CHU Lille, Institut Pasteur de Lille, U1011 – EGID, Lille, France; ^4^Université d’Artois EA 2465, Laboratoire de la Barrière Hémato-Encéphalique (LBHE), Lens, France; ^5^Université Lille Nord de France, Unité Environnement Périnatal et Croissance EA 4489, Équipe dénutritions maternelles périnatales, Université Lille 1, Villeneuve-d’Ascq, France

**Keywords:** opioid peptides, hemorphins, intestinal barrier, blood–brain barrier, Caco-2 model, brain-like endothelial cell model, claudin-4, cAMP

## Abstract

A qualitative study is presented, where the main question was whether food-derived hemorphins, i.e., originating from digested alimentary hemoglobin, could pass the intestinal barrier and/or the blood–brain barrier (BBB). Once absorbed, hemorphins are opioid receptor (OR) ligands that may interact with peripheral and central OR and have effects on food intake and energy balance regulation. LLVV-YPWT (LLVV-H4), LVV-H4, VV-H4, VV-YPWTQRF (VV-H7), and VV-H7 hemorphins that were previously identified in the 120 min digest resulting from the simulated gastrointestinal digestion of hemoglobin have been synthesized to be tested in *in vitro* models of passage of IB and BBB. LC-MS/MS analyses yielded that all hemorphins, except the LLVV-H4 sequence, were able to cross intact the human intestinal epithelium model with Caco-2 cells within 5–60 min when applied at 5 mM. Moreover, all hemorphins crossed intact the human BBB model with brain-like endothelial cells (BLEC) within 30 min when applied at 100 µM. Fragments of these hemorphins were also detected, especially the YPWT common tetrapeptide that retains OR-binding capacity. A cAMP assay performed in Caco-2 cells indicates that tested hemorphins behave as OR agonists in these cells by reducing cAMP production. We further provide preliminary results regarding the effects of hemorphins on tight junction proteins, specifically here the claudin-4 that is involved in paracellular permeability. All hemorphins at 100 µM, except the LLVV-H4 peptide, significantly decreased claudin-4 mRNA levels in the Caco-2 intestinal model. This *in vitro* study is a first step toward demonstrating food-derived hemorphins bioavailability which is in line with the growing body of evidence supporting physiological functions for food-derived peptides.

## Highlights

- Food-derived hemorphins cross intestinal barrier in vitro.
- Food-derived hemorphins cross human blood–brain barrier (BBB) in vitro.
- New hemorphin sequences behave as opioid receptor (OR) agonists in Caco-2 cells.
- Food-derived hemorphins modulate claudin-4 mRNA levels in Caco-2 cells.
- Naloxone behaves as OR agonist in Caco-2 cells.

## Introduction

Hemorphins are a group of opioid peptides encrypted in the beta-chain of hemoglobin, in a conserved region between bovine and human hemoglobin. Hemorphins, from endogenous or food-derived hemoglobin, are found in many different tissues and species (1–3) and many bioactivities have been uncovered for these hemorphins, notably in blood pressure regulation (4–6) and cognitive functions (7). They were first shown to interact with opioid receptors (ORs) (8, 9) that earned them the name “hemorphin,” and explain other activities like intestinal peristalsis (10, 11), bladder contraction, inflammation, and pain modulation (12–14). Our laboratory is interested in the fate of alimentary or digested hemoglobin protein and particularly in the derived bioactive peptides thereof in relation to food intake regulation. We have previously identified five hemorphins from the simulated gastrointestinal digestion of hemoglobin. This simulated gastrointestinal digestion has been validated (15) and is now routinely used to identify and highlight bioactivities of food-derived peptides. These five hemorphins that were produced by and resistant to gastrointestinal digestion have been studied for their effects at the intestinal lumen level in relation to gut hormones synthesis and release and DPP-IV (CD26) regulation (16). Among these five hemorphins (Table 1), three have an N-terminal extension, LLVV-, LVV-, and VV-, one has a C-terminal extension, -QRF, and one has both an N-terminal, VV- and C-terminal -QRF, extension of the tetrapeptide core YPWT that has been demonstrated to bind to OR.

**Table 1:** Passage of five hemorphins identified in the intestinal fraction of (*in vitro*) gastrointestinal digests of hemoglobin, through the intestinal barrier (IB) (caco-2 model) and blood–brain barrier (BBB) (co-culture model of hematopoietic CD34+-derived endothelial cells with brain pericytes) *in vitro*.

	Intestinal barrier			BBB
Hemorphins applied	Apical		Basal	Basal
LLVV-H4	LLVV-H4, LVV-H4, VV-H4, H4	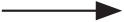	H4	H4, VV-H4, LVV-H4, LLVV-H4
LVV-H4	LVV-H4, VV-H4, H4	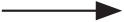	H4, VV-H4, LVV-H4	H4, VV-H4, LVV-H4
VV-H4	VV-H4, H4	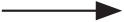	H4, VV-H4	H4, VV-H4
VV-H7	VV-H7, VV-H4, H7, H6	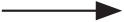	H4, VV-H7, H7, H3	H4, VV-H7, VV-H4, H7, H3, H5
H7	H7, H6, H4, H3	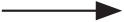	H4, H7, H3	H4, H7, H3, H5

*Five hemorphins, LLVV-H4 (LLVV-YPWT), LVV-H4 (LLVV-YPWT), VV-H4 (VV-YPWT), VV-H7 (VV-YPWTQRF), and H7 (YPWTQRF) have been applied separately in the apical side of epithelium in each passage test. This table sums up the presence of the intact peptides or their fragments in each compartment at the end of the 120 min passage test*.

Opioid receptors are involved in many aspects of food intake regulation *via* central effects (17). Recently, peripheral ORs have also been involved in these regulations and a gut-brain loop has been described with a crucial role of the portal vein mu-OPs (18). It was thus proposed that opioid peptides originating from the digestion and absorption of dietary proteins would interact with the OR located in the portal vein and trigger a gut-brain loop mediating high-protein diet-induced satiety.

Given hemorphins size and evidence from the literature for other exorphins (19), it is likely that the passage of these peptides belongs to the paracellular transport mode involving effects on tight junction (TJ). Moreover, there is some literature implicating opioids in intestinal permeability disorders and TJ regulation. There are known deleterious effects of mu-OR agonists on the gut barrier and immune function in pain-treated patients or drug abusers (20, 21). In mice, it was shown that morphine treatment is associated with disruption of TJ organization (22). TJ proteins are an ensemble of protein families that seal the paracellular space between epithelial cells. TJ proteins include transmembrane proteins, such as occludin and claudin families, and scaffolding proteins, such as zonula occludens-1 (ZO-1) and -2 (ZO-2).

Hence, we sought, by using two *in vitro* models, to demonstrate that food-derived hemorphins, intact or as fragments retaining OR-binding capacity, pass the intestinal and the BBBs. For the IB, Caco-2 was used as a well recognized and widely used model of human intestinal epithelium (23, 24). Indeed, according to the Biopharmaceutics Classification System, there is a high correlation between Caco-2 cell permeability coefficients and fractional absorption values in humans (25). Moreover, especially interesting in the present study where the BBB passage has also been tested, the Caco-2 model revealed to be predictive of oral bioactivity and of BBB permeability (26), although another study concluded to a poor correlation between Caco-2 cell data and *in vivo* BBB transport (27). Several BBB models have been described over the past 40 years and are currently used in different research groups to analyze different aspects of BBB biology and drug targeting. The human brain-like endothelial cells (BLEC) co-culture model recently described (28) was chosen in this study. It expresses TJ and transporters typically observed in brain endothelium, displays most *in vivo* BBB properties and thus could be used for both mechanistic studies and as a screening tool for CNS-compound permeability studies in human (29, 30).

We report here the results of both *in vitro* intestinal and BBB passage tests, analyzed by LC-MS/MS, of five food-derived hemorphins and preliminary results of hemorphins impact on TJ proteins of the IB.

## Materials and Methods

### Chemicals

Dulbecco’s modified eagle’s medium (DMEM) with high glucose (4.5 g L^−1^), fetal bovine serum (FBS), Dulbecco’s phosphate buffered saline, Hank’s balanced salt solutions (HBSS), lucifer yellow (LY), Bestatin, thiorphan, naloxone, DAMGO, forskolin (FK), and Whatmann GF/B glass microfiber filter were purchased from Sigma-Aldrich (Steinheim, Germany). l-Glutamine and penicillin-streptomycin were from Pan-Biotech (Aidenbach, Germany). Cell culture inserts with polycarbonate membranes (3 µm pore size; 4.2 cm^2^ surface area and 0.4 µm pore size; 1.12 cm^2^ surface area) and companion plates were purchased from Corning (Boulogne-Billancourt, France). H3-naloxone and the scintillation liquid Optiphase HiSafe 2 was obtained from PerkinElmer (Courtaboeuf, France). For the determination of intracellular cAMP, the Mouse/Rat cAMP Parameter Assay Kit was used (R&D Systems, Bio-Techne, Lille, France). Protein dosage assays were performed with the Sigma QuantiPro BCA Assay Kit (Sigma-Aldrich, Steinheim, Germany) or the Pierce BCA Protein Assay Kit (Thermo Fisher Scientific, Saint-Aubin, France). Reagents for the preparation and performance of quantitative real-time PCR (qPCR) were TRI Reagent^®^ (Sigma-Aldrich, Steinheim, Germany), RevertAid H Minus First Strand cDNA Synthesis Kit and the Power SYBR Green PCR Master Mix (Thermo Fisher Scientific, Saint-Aubin, France). Specific oligonucleotides were purchased from Eurogentec (Seraing, Belgium). Synthetic peptides were purchased from GeneCust (Dudelange, Luxemburg).

### Cell Culture

For the cAMP determination assay and the gene expression study, the cell line used was the Caco-2 cell line purchased from Sigma-Aldrich (Steinheim, Germany). Cells were grown at 37°C, 5% CO_2_ atmosphere in Dulbecco’s modified Eagle’s Medium (DMEM, 4.5 g L^−1^ glucose) supplemented with 10% FBS, 2 mM l-glutamine, 100 U mL^−1^ penicillin, and 100 µg mL^−1^ streptomycin (complete DMEM) until use. Caco-2 from passages 34–37 were used. For the transport studies, the cell lines used and the culture protocols conditions are described in detail in dedicated subsections below.

### Transport Studies

#### *In Vitro* Human IB Model

The human intestinal cell line used for the transport experiments was the Caco-2/TC7 clone, a gift from Dr. Monique Rousset (UMRS 872, INSERM, Paris). Cells were routinely grown as previously described (31). For the experiment, they were seeded at the density of 60 × 10^3^ cells cm^−2^ into cell culture inserts in 6-well culture plates and grown for 21 days.

The day of the trans-intestinal transport test, growth media were removed, and cells were first rinsed with the transport medium, HBSS-Hepes buffer supplemented with CaCl_2_ (2.5 mM final) and MgCl_2_ (0.5 mM final) in the apical (1 mL) and basolateral chambers (2.5 mL), then pre-incubated for 30 min with the transport medium. All incubations were performed at 37°C, 10% CO_2_ atmosphere. To control the integrity of the Caco-2/TC7 cell barrier, the paracellular transport marker lucifer yellow (LY, 100 µM final) was applied apically into each well and its transport into the basolateral chamber was monitored by spectrofluorescence. Each hemorphin was diluted for a final concentration of 5 mM in transport medium-LY and applied to the apical chamber at t0. In parallel, control wells were incubated with transport medium-LY only and all conditions were tested in triplicate. At t5, 15, 30, and 60 min, 100 µL samples were collected in the basal chamber and replaced by the same volume of transport medium. At t120 min, all remaining media in basal and apical chambers were collected and all samples were stored at −80°C. The rate of LY transport was determined by fluorescence readings (ex 485/em 530 nm) at each time point on the spectrofluorometer Safas Xenius XC (Safas Monaco, Monaco, France) and calculating the apparent permeability coefficient (Papp) as follows: (ΔQ/Δt) × (Vb/(A × C0)), where ΔQ is the change in LY concentration in the basal chamber (μM); Δt is the transport duration (s); Vb is the volume in the basal chamber (mL); A is the surface area of the insert/transwell membrane (cm^2^); and C0 is the initial concentration of LY applied to the apical chamber (μM). Papp is measured in cm s^−1^ and the Caco-2/TC7 cell barrier for each sample was considered intact if Papp <1 × 10^−6^ cm s^−1^.

#### *In Vitro* Human BBB Model: BLEC Model

The brain-like endothelial cell model has previously been described in detail (28). All the sample donors had given their written informed consent, in compliance with French legislation and the 2013 version of the Declaration of Helsinki. The sample collection was approved by the local investigational review board (Bethune Maternity Hospital Béthune, France). Briefly, this human model consists in isolating CD34+ cells from umbilical cord blood. These cells are then cultured in endothelial cell medium supplemented with 20% FCS for 15–20 days. After this period, cells are differentiated into endothelial cells. After a trypsinization step, they are seeded on matrigel coated-transwell inserts in 12-well culture plates, and subsequently cultured with brain pericytes, isolated from bovine brain capillaries. After 6 days of co-culture, human endothelial cells acquire the BBB phenotype, and are then named BLECs.

The day of the transport test, the same procedure as for the intestinal passage assay was applied except that the transwell inserts were transferred to a new 12-well culture plate at each time point, 30, 60, 90, and 120 min. All incubations were performed at 37°C, 5% CO2 atmosphere with slight agitations. The transport medium consisted in Ringer-Hepes solution (150 mM NaCl, 5.2 mM KCl, 2.2 mM CaCl_2_, 0.2 mM MgCl_2_⋅6H_2_O, 6 mM NaHCO_3_, 5 mM HEPES, pH 7.4). Each hemorphin was diluted for a final concentration of 100 µM in transport medium-LY and applied to the apical chamber (0.5 mL; 1.5 mL transport medium in basal chamber) at t0. A test was performed without cells to insure that the filter membrane was not preventing the passage of the peptides. At each time point, 200 µL samples were collected in the basal chamber for the LY assay (measured with Synergy H1, BioTek) and the remaining 1.3 mL were collected at 30 min and frozen at −80°C for further LC-MS/MS analyses.

### LC-MS/MS

LC-MS/MS analysis was performed on a UFLC-XR device (Shimadzu, Kyoto, Japan) coupled to a QTRAP^®^ 5500 MS/MS hybrid system triple quadrupole/linear ion trap mass spectrometer (AB Sciex, Foster City, CA, USA) equipped with a Turbo VTM ion source. Instrument control, data acquisition, and processing were performed using Analyst 1.5.2 software. The reverse phase liquid chromatography separation was carried out on a Kromasil C18 column (100 × 2.1 mm, 3.5 µm) with guard cartridge from AIT-France (Houilles, France). The injection volume was 2 µL. Elution was performed at a flow rate of 200 µL min^−1^ with water-formic acid 0.1% as eluent A and acetonitrile-formic acid 0.1% as eluent B. The injection duty cycle was 10 min, starting with a 1 min plateau with 25% B, followed by a linear gradient from 25 B to 60% B in 4 min, a 1 min plateau with 60% B, 1 min linear gradient from 60 to 25% B, and 3 min at 25% B to recondition the column. MS analysis was carried out in positive ionization mode. The ion source parameters were optimized and set as follows: ion spray voltage, 5,500 V; nebulizer gas (air) and curtain gas (nitrogen) flows, 50 and 25 psi, respectively; source temperature, 550°C with the auxiliary gas flow (air) set at 50 psi; declustering potential (DP), 100 V; and collision cell exit potential, 25 V. The mass spectrometer was operated at a unit resolution for both Q1 and Q3 with a dwell time of 90 ms in each transition.

The presence of each intact hemorphin and potential fragments were tracked by a combination of multiple reaction monitoring (MRM) and pseudo-MRM transitions. V-H4 and N-extended-H3 fragments were not searched because they probably present poor OR ligand capacity (32) and they have not been reported as present in body fluids or tissues.

### Radiobinding

The potential binding of each opioid peptide/hemorphin on OPs was assessed in a radiobinding competition test with H3-naloxone as the tritiated-specific ligand on rat brain membrane preparation. The protocol used follows the one described in Garreau et al. (9) with some modifications. Briefly, rat brains were homogenized in 50 mM Tris–HCl added with 240 mM sucrose, 5 mM MgCl_2_, and 2 mM EDTA and centrifuged first at 1,000 *g* for 5 min at 4°C. Supernatants were then centrifuged at 30 × 10^3^ *g* for 30 min at 4°C and the resulting pellets were solubilized in the homogenization buffer. Protein dosage was performed with the Sigma QuantiPro BCA Assay Kit. Serial dilutions of each peptide or naloxone as positive control were realized in 50 mM Tris–HCl buffer pH 7.4 supplemented with 2% bovine serum albumin and incubated in 0.9 mg mL^−1^ of rat brain membrane preparation added with the proteases inhibitors bestatin (10 µM final) and thiorphan (0.1 µM final), and 1 nM final H3-naloxone for 30 min at 25°C. Non-specific binding was determined by adding an excess of naloxone (5 × 10^−6^ M final) instead of the peptide or naloxone dilutions. All conditions were run in duplicates. At the end of the incubation, separation of bound and unbound samples was realized by vacuum filtration through glass microfiber GF/B filters. Filters were inserted into scintillation vials, added with 3 mL Optiphase HiSafe 2 scintillation liquid, and radioactivity was counted on a beta-counter (Hidex 300 SL, Sciencetec, Villebon-sur-Yvette, France). Non-specific binding was subtracted from all values and specific binding was expressed as a percentage of total-specific binding (dpm). Competition binding curves were plotted in GraphPad Prism v6.01 and ED_50_ were determined by non-linear regression analysis.

### cAMP Determination Assay

The effect of the five opioid peptides/hemorphins on the cAMP production was assessed *in vitro* in Caco-2 cells. Each hemorphin was applied on Caco-2 cells stimulated or not by FK, an activator of the cAMP pathway, in order to evaluate the ability of the peptides to decrease intracellular cAMP elevation induced by FK. Cells were seeded at 1 × 10^5^ cells/well in 24-well plates and grown for 7 days in complete culture medium. The medium was renewed 24 h before the test and replaced by the incubation medium, DMEM supplemented with 1% l-glutamine (DMEM-l-glu) 30 min prior to the test start. Each hemorphin was applied at 50, 500, or 5,000 µM (FK-stimulated condition) and 50 or 500 µM (non-stimulated condition) for 15 min at 37°C. The specific OR agonist DAMGO (1 and 100 nM) and the OR antagonist naloxone (1 µM) were tested in the same conditions. To verify that the hemorphin-induced effect on cAMP production was mediated through the activation of OPs, cells were pre-incubated for 30 min with 1 µM naloxone in DMEM-l-glu prior to the incubation with each opioid peptide (500 µM) or DAMGO (100 nM) in DMEM-l-Glu-FK in the same conditions as previously. All conditions were run in triplicate. At the end of the 15 min incubation, cells were immediately rinsed three times with PBS and plates were stored at −80°C until intracellular cAMP levels determination with the Mouse/Rat cAMP Parameter Assay Kit according to the manufacturer’s guidelines. cAMP levels were normalized by the total protein concentration determined by the Pierce BCA Protein Assay Kit for each sample and expressed as a percentage of the reference condition FK only.

### Gene Expression Analysis

The impact of opioid peptides on claudin-4 gene expression in Caco-2 cells was evaluated by qPCR. Caco-2 were seeded at 80 × 10^3^ cells cm^−2^ in cell culture inserts into 6-well culture plates and first cultured in complete DMEM for 3 weeks with media changes (basal and apical) every 2–3 days. 100 µM of each hemorphin in DMEM-l-glu or incubation buffer only were applied in triplicate in the apical compartment for 24 h at 37°C, 5% CO_2_. At the end of the incubation time, cells were rinsed two times in PBS and scrapped in TRI Reagent. Total RNA was further isolated and processed according to the protocol described in Caron et al. (15). qPCR analyses were performed with specific oligonucleotides for claudin-4: forward (F) 5′-CCACTCGGACAACTTCCCAA-3′ and reverse (R) 5′-ACTTCCGTCCCTCCCCAATA-3′, and peptidylprolyl isomerase (PpiA): (F) 5′-TGCTGACTGTGGACAACTCG-3′ and (R) 5′-TGCAGCGAGAGCACAAAGAT-3′ and the Power SYBR Green PCR Master Mix on a StepOne™ Plus system (Applied BioSystems, Life Technologies). mRNA fold induction was calculated according to the 2^−ΔΔCt^ method (ΔΔCt method, Applied Biosystems User Bull. #2 Dec. 97) using PpiA as internal control (reference gene) and expressed as a percentage of the control group induction.

### Statistical Analysis

Data presented are mean ± SD. Statistical analysis was conducted in GraphPad prism v6.1. One-way ANOVA was applied with Tukey or Dunnett’s test for *post hoc* analyses. For comparison of the cAMP levels, a two-way ANOVA was used to consider the different concentrations used, followed by a Dunnett’s test for multiple comparisons. To assess the effect of the naloxone treatment in the complementary cAMP assay, a two-way ANOVA was used, and each hemorphin effect was compared to its corresponding naloxone treatment by a Sidak test. The Dunnett’s *post hoc* test was used for DAMGO comparisons. Significance level was set at *p* < 0.05.

## Results

### IB Passage

**Figure 1:**
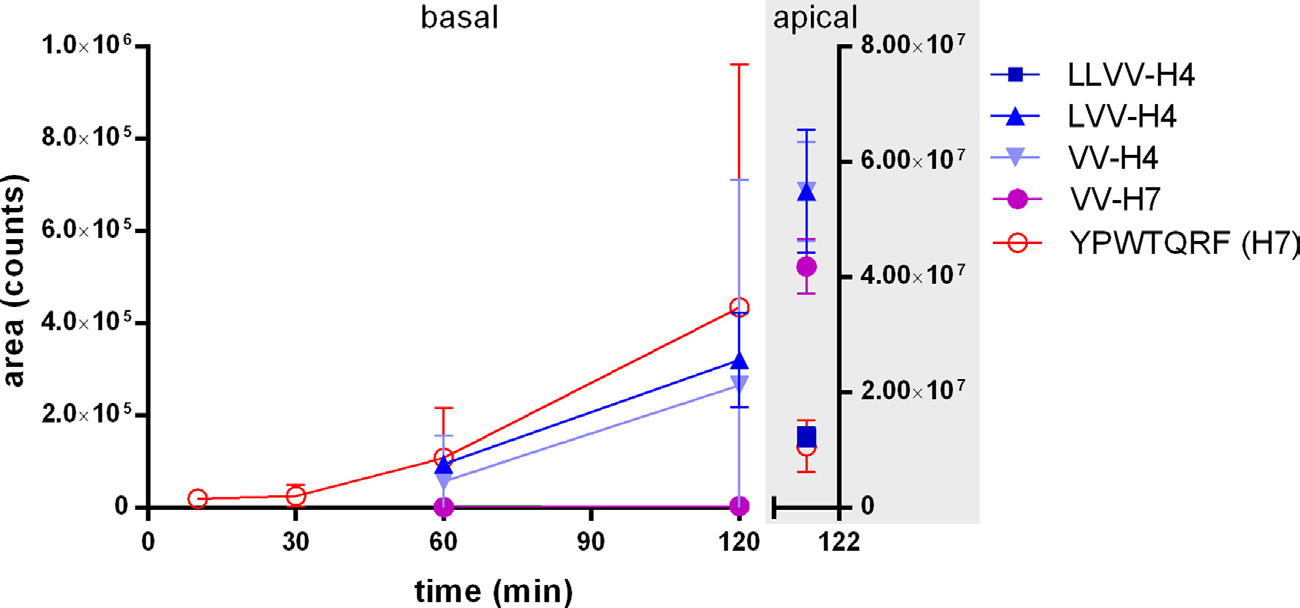
Appearance kinetics of the five hemorphins intact in the basal side of Caco-2 monolayer as an intestinal barrier (IB) passage model. The passage of the five hemorphins was tested in the Caco-2-transwell model of the IB. Each peptide was separately applied at 5 mM in the apical chamber of a transwell and the appearance of the intact peptide was monitored by LC-MS/MS with multiple reaction monitoring in the basal chamber at five time points. The graph also shows the remaining intact peptides in the apical chamber after 2 h incubation. Data are expressed as mean ± SD (*n* = 3).

In first analysis, the samples collected at each time point in the basal compartment below the Caco-2 cells layer modeling the IB were analyzed by LC-MS/MS with MRM to detect the whole peptide sequences. The analysis revealed that LVV- and VV-H4 as well as H7 were clearly able to cross intact the cell monolayer, whereas VV-H7 was found in traces and LLVV-H4 was not detected (Figure 1). H7 displayed the most rapid passage as it appeared basally as soon as 5 min incubation and its basal concentration increased faster. LVV- and VV-H4 appeared in the 60 min samples and behaved similarly. All peptides except the LLVV-H4 sequence were still found intact and abundant in the apical side after 120 min incubation (Figure 1).

**Figure 2:**
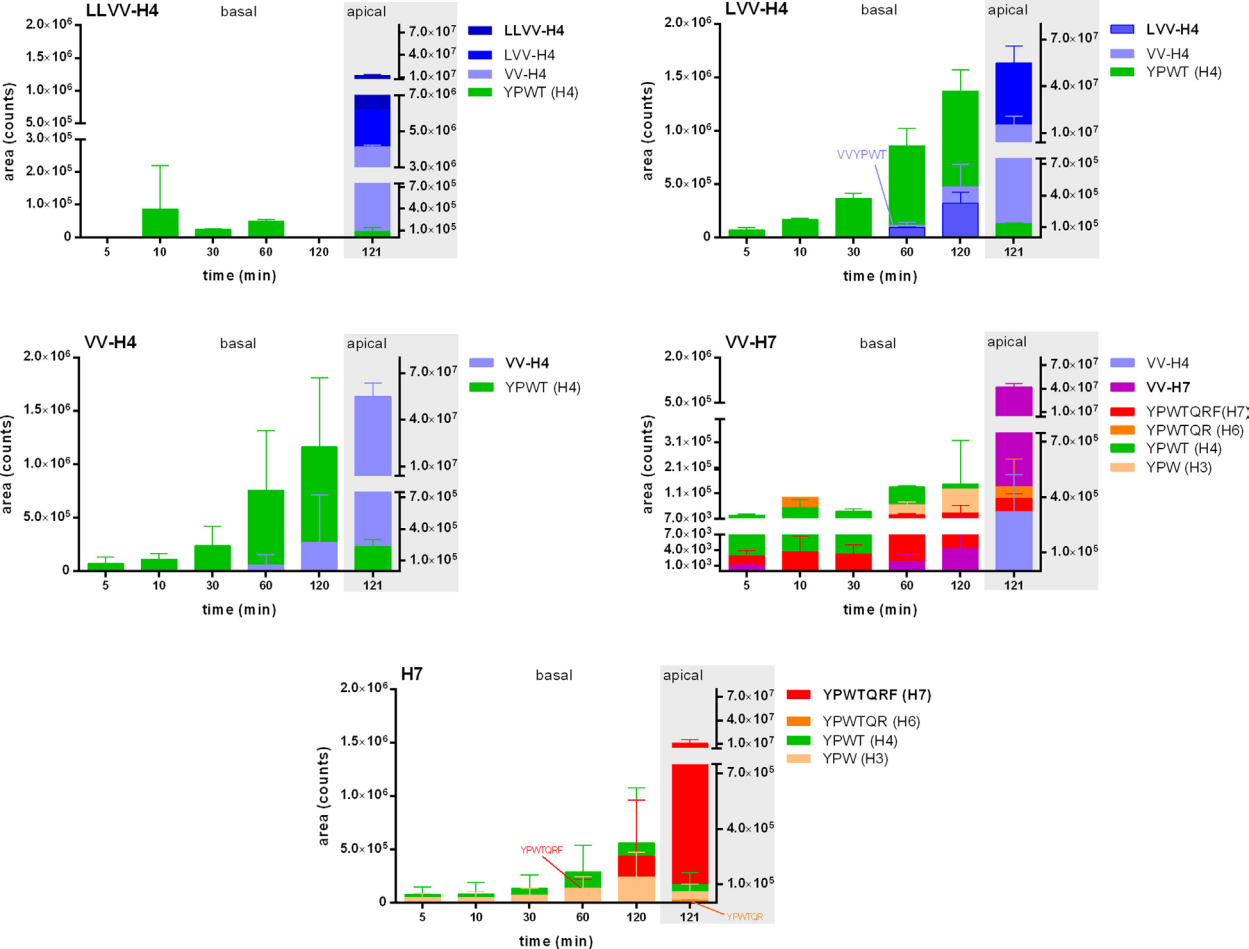
Release kinetics of the five hemorphins fragments in the basal side of a Caco-2 monolayer as an intestinal barrier passage model. The metabolism of the five hemorphins was evaluated by monitoring the apparition of peptide fragments issued from the opioid peptides by selected ion monitoring analyses in the basal chamber at five time points. The presence of these fragments was also investigated in the apical chamber after the 2 h incubation to determine whether these fragments were the results of the passage through the Caco-2 monolayer. Data are expressed as mean ± SD (*n* = 3).

Nevertheless, in addition to the intact peptides, potential fragments all comprising the tripeptide YPW were then searched in each basal sample by LC-MS/MS with pseudo-MRM. Expectedly, the peptides with an N-terminal extension were hydrolyzed and gave the other peptide sequences under investigation (e.g., in the well where LLVV-H4 was applied, LVV- and VV-H4 were found as well). However, although LVV- and VV-H4 were able to cross the cell barrier when applied alone, they were not found in the basal side of the LLVV-H4 wells, where they were produced apically. Interestingly, for all tested peptides the most abundant fragment identified is the tetrapeptide core YPWT (Figure 2).

**Figure 3:**
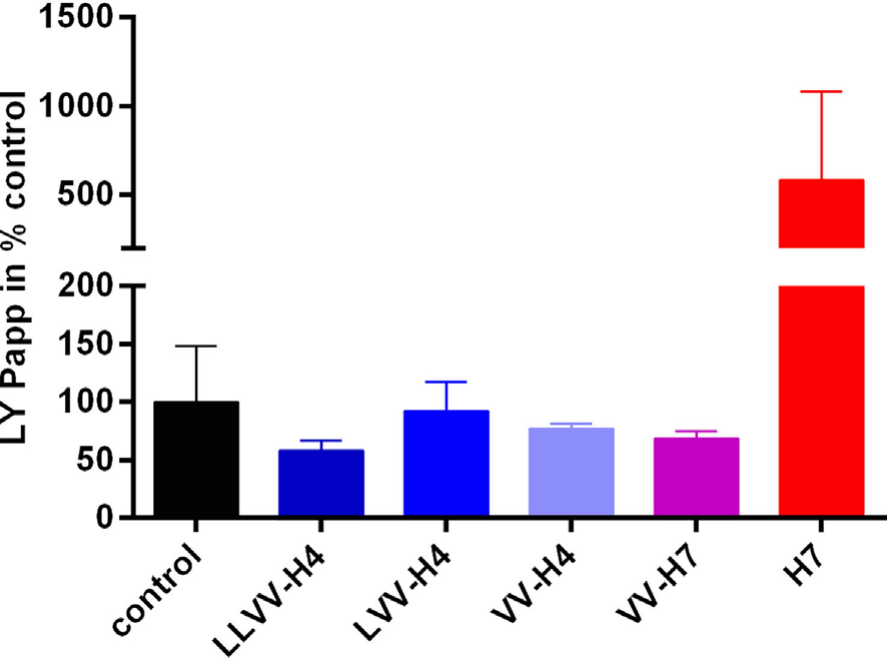
Lucifer yellow (LY) permeability across the intestinal barrier model in presence of each hemorphin. The marker of paracellular transport LY was applied together with each hemorphin on the apical side of the transwell and its passage into the basolateral chamber was monitored by spectrofluorescence. Collecting basal samples at different time points allowed calculation of the apparent permeability coefficient (Papp) of LY which, when below 1e^−006^ cm s^−1^ is commonly indicative of the cell barrier integrity.

Results from the LY passage monitoring indicated that the integrity of the cell monolayer was not altered throughout the 120 min incubation (Figure 3). Papp values calculated were all below 1 × 10^−6^ cm s^−1^ which is a commonly accepted threshold for barrier integrity. However, though not statistically significantly different from the control value (as assessed by Dunnett’s *post hoc* analysis), peptides LY Papp values seemed to correlate with the ability of the hemorphins to cross the cell barrier. Indeed, peptides with Papp(LY) values similar or above the control one showed the most passage. This was the most striking for H7 for which Papp(LY) was almost six times higher than control one (7.632e−007 ± 6.504e−007 > 1.310e−007 ± 6.308e−008 cm s^−1^, *p* = 0.0503), whereas LLVV-H4 and VV-H7 which did not or barely crossed the barrier displayed Papp(LY) decreased compared to control (non-significant).

### BBB Passage

**Figure 4:**
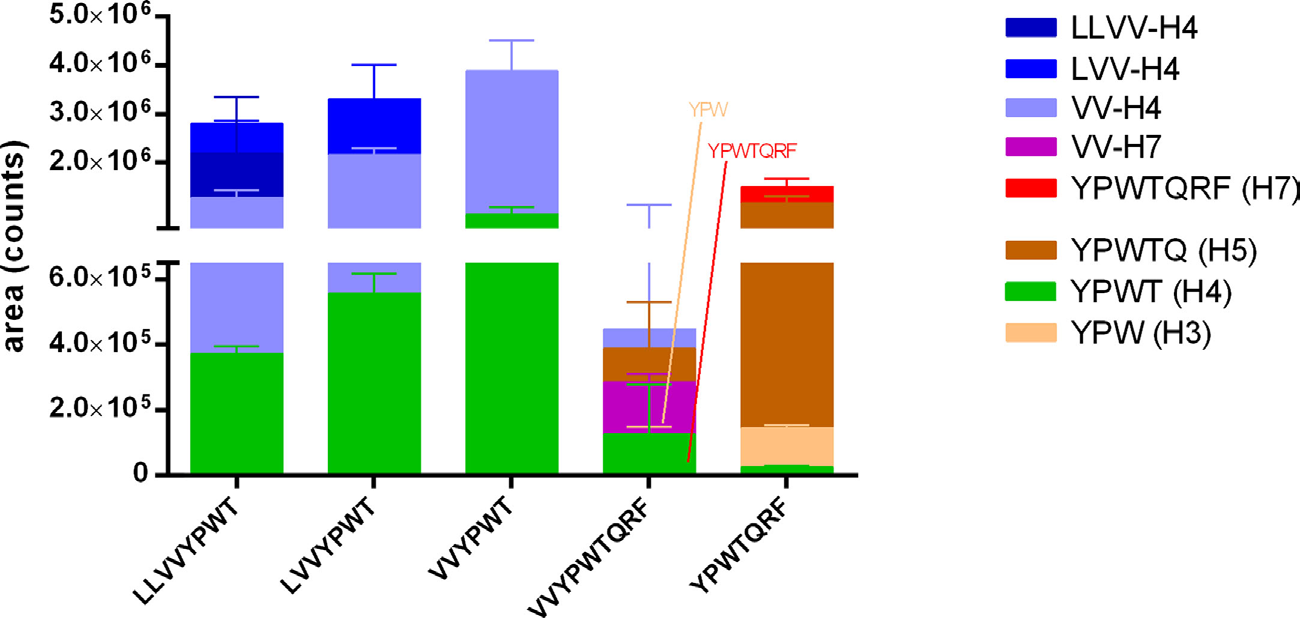
Passage of the five hemorphins intact or their fragments across the brain-like endothelial cell blood–brain barrier (BLEC) model. The passage of the five hemorphins was tested in the BLEC model (co-culture of hematopoietic CD34+-derived endothelial cells with bovine pericytes). Each peptide was separately applied at 100 µM in the apical chamber (“blood” side) of a transwell and the presence of the intact peptides and potential fragments thereof in the basal chamber (“brain” side) was investigated by a combination of LC-MS/MS with multiple reaction monitoring (MRM) and pseudo-MRM analyses after 30 min incubation. Data are expressed as mean ± SD (*n* = 3).

The same LC-MS/MS analyses were applied on the samples collected during the test of passage of the hemorphins across the co-culture layer modeling the BBB. The analyses revealed that all peptides were able to cross intact this cell barrier (Figure 4). In comparison to the IB model, the transport was faster and more efficient, since all hemorphins were detected basally already at 30 min of incubation and the passage occurred despite a much lower initial apical concentration, i.e., 100 µM in the BBB model vs 5 mM in the IB model.

The search for the peptides fragments yielded partially the same results. The same fragments were identified except the fragment YPWTQ which was only detected in the basal VV-H7 and H7 samples of the BBB co-culture model. Moreover, the fragment YPWT was also found basally in all samples. In contrast to the results of the IB test, YPWT was not the main fragment and longer ones were also found in the basal compartment.

**Figure 5:**
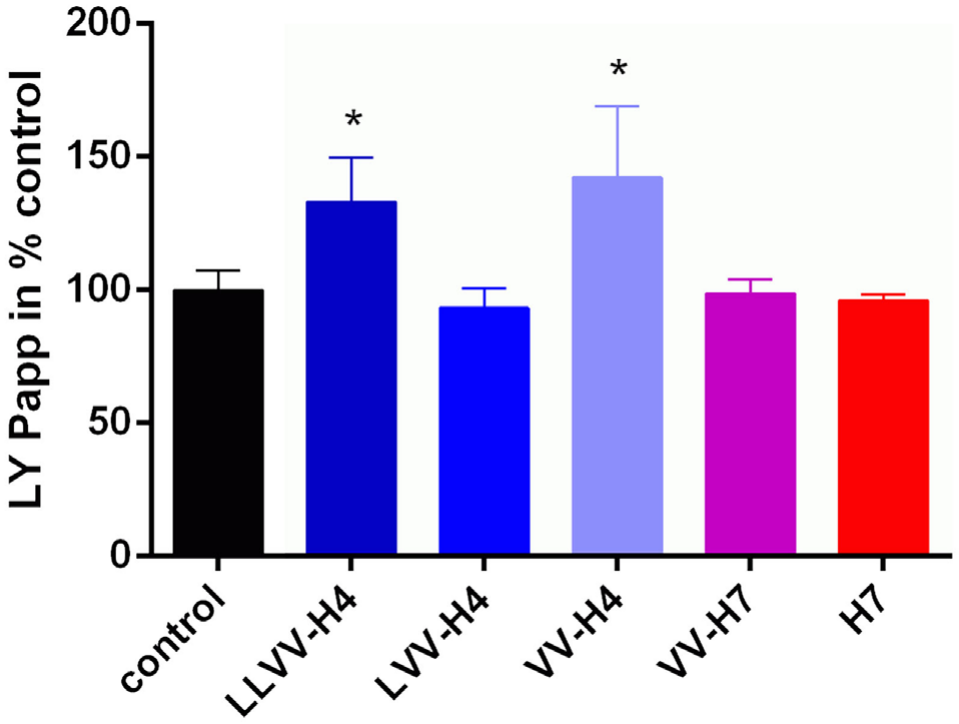
Lucifer Yellow (LY) permeability across the blood–brain barrier model in presence of each hemorphin. The marker of paracellular transport LY was applied together with each hemorphin on the apical side of the transwell and its passage into the basolateral chamber was monitored by spectrofluorescence. Collecting basal samples at different time points allowed calculation of the apparent permeability coefficient (Papp) of LY which, when below 1e^−006^ cm s^−1^ is commonly indicative of the cell barrier integrity.

With regards to the LY permeability, LLVV-H4 and VV-H4 significantly increased Papp(LY) compared to control (Dunnett’s *post hoc* analysis: 133.2 ± 16.44%, *p* = 0.0446 and 142.2 ± 26.71%, *p* = 0.0106, respectively) without affecting the cell barrier integrity (Figure 5). This was reflected by the Papp values that were all below 1 × 10^−6^ cm s^−1^ which is a commonly accepted threshold for barrier integrity.

### OP Binding

**Figure 6:**
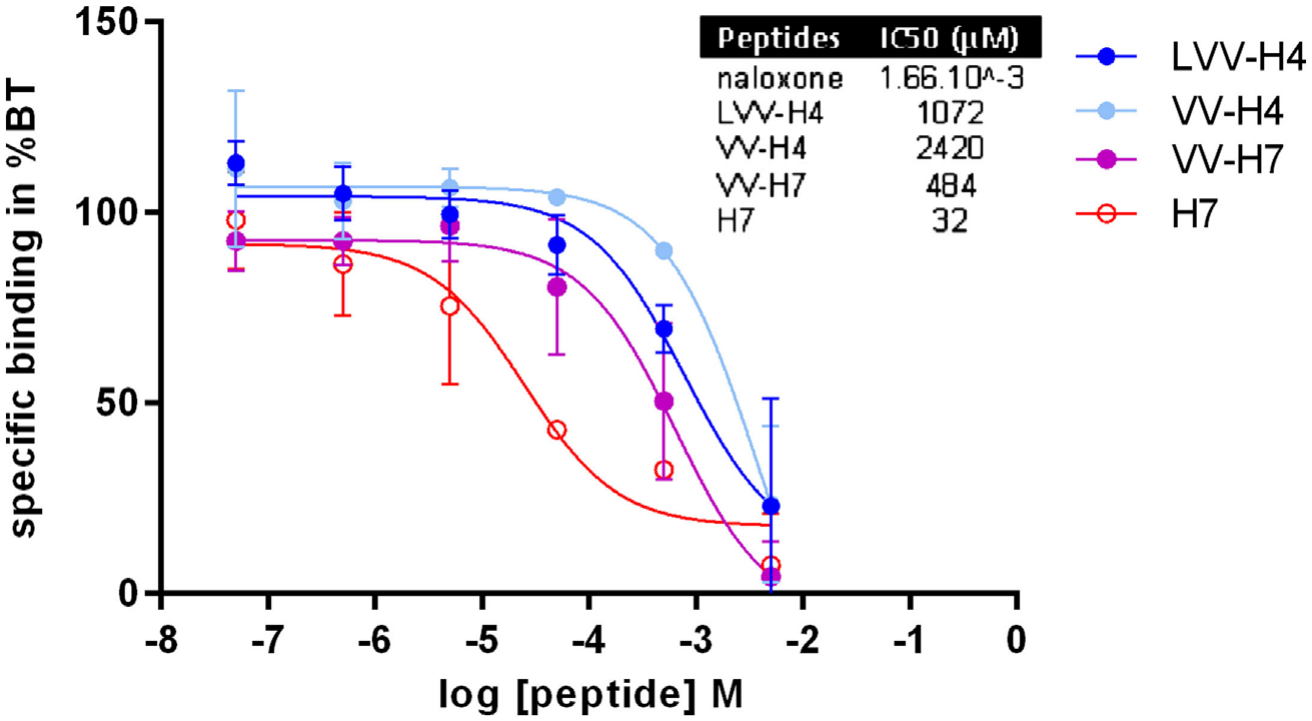
Determination of hemorphins binding to rat brain membranes opioid receptors (OPs). The ability of the hemorphins/opioid peptides to bind to OPs was assessed in a radiobinding assay *in vitro* by competition with H3-naloxone (non-selective OP antagonist) on rat brain membrane preparation. Six concentrations ranging from 5 × 10^−8^ to 5 × 10^−3^ M were tested in duplicates and the results were expressed as a percentage of total binding without opioid peptides. Inset: dose–response curves were analyzed in GraphPad Prism 6 by non-linear regression from which IC_50_ were interpolated. Data presented are mean (average of two separate assays) ± SD.

The determination of the OP-binding capacity of each hemorphin was performed on rat brain membrane preparation. The binding capacity of peptide LLVV-H4 could not be determined in the same conditions as for the other peptides, since its solubility was poor in the incubation buffer at concentrations higher than 5 × 10^−5^ M. Especially because of the method of separation of the bound and unbound ligands on glass microfiber filters; we suspected that the peptide dilution clogged the filter. Nevertheless, at least for the 10^−10^ to 10^−4^ M range (data not shown) LLVV-H4 was not able to compete with the specific OR tritiated antagonist. In contrast, all other tested hemorphins were able to decrease the fixation of the ^3^H-naloxone on OPs in a dose-dependent manner. Inhibition of the antagonist binding on OR was efficient in the 5 × 10^−7^ to 5 × 10^−3^ M range for H7 and in the 5 × 10^−5^ to 5 × 10^−3^ M range for the other hemorphins (Figure 6). Thus, except for H7, the binding curves display a steep decrease within 2 log units of hemorphins concentrations suggesting a competition for a single binding site. H7 binding curve displayed a shallower decrease on nearly 4 log units that could be indicative of a competition for more than one binding site.

As illustrated by the competitive binding curve and the determination of IC_50_, the affinity of the hemorphins ranked as follows from the most affinity to the least: H7 > VV-H7 > LVV-H4 > VV-H4 (Figure 6 inset). The presence of the N-terminal extension impaired or reduced binding (compared to the C-terminal only extension), since peptides with an N-terminal extension only displayed the highest IC_50_ and the peptide VV-H7 with both N- and C-terminal extensions presented an intermediary IC_50_.

### cAMP Pathway Activation

**Figure 7:**
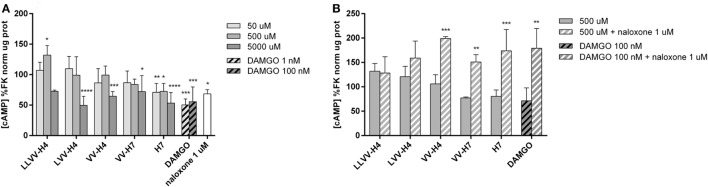
Determination of intracellular cAMP in forskolin (FK)-stimulated Caco-2 cells after incubation with the five hemorphins. **(A)** The ability of the hemorphins/opioid peptides to act on the cAMP pathway was evaluated *in vitro*. Caco-2 cells were incubated with or without increasing concentrations of each hemorphin, DAMGO, a known specific OP agonist and naloxone, a known OP antagonist, in FK-supplemented medium for 15 min at 37°C. Intracellular cAMP levels were determined by ELISA, normalized by the total protein concentration and expressed as a percentage of the reference group FK. Data presented are mean ± SD (average of two assays performed in triplicate; *n* = 6). *Statistically different from control; *p < 0.05, **p < 0.01, ***p < 0.005. **(B)** To determine whether the effect of the hemorphins was mediated through OPs, the same assay was performed with or without naloxone (1 µM) in the same incubation medium. Data presented are mean ± SD (*n* = 3). *Statistically different from control; **p < 0.01, ***p < 0.005.

In order to precise the OR agonist, antagonist or mixed nature of the hemorphins, their effect on the accumulation of intracellular cAMP in Caco-2 cells upon stimulation with an activator of adenylate cyclase, FK, was investigated. OR agonists have a known inhibitory action on adenylate cyclase with a consecutive reduction of intracellular cAMP formation (33). Measurement of intracellular cAMP under different concentrations of each hemorphin in FK-stimulated cells did not show a clear dose effect. The highest tested concentration of 5 mM did induce a significant decrease in FK-stimulated cAMP concentration compared to control levels for all sequences, except LLVV-H4, and is indicative of an agonist-like behavior of the hemorphins (Figure 7A). The highest reductions were achieved by LVV-H4, 50% (49.57 ± 14.79% of FK levels, *p* < 0.001) and H7, 47% (53.24 ± 17.41% of FK levels, *p* < 0.001), while VV-H4 induced a reduction of 35% (64.56 ± 7.67% of FK levels, *p* = 0.0009) and VV-H7 of 28% (72.01 ± 26.42% of FK levels, *p* = 0.0104). Notably, H7 was able to inhibit FK-induced cAMP elevation (of about 30%) at the two other lower concentrations of 500 and 50 µM (72.38 ± 13.25%, *p* = 0.0117 and 70.96 ± 14.58% of FK levels, *p* = 0.0075, respectively). Thus, 5 mM of LVV-H4 and H7 produced a decrease in the same size range, about 50%, as following the specific MOR agonist DAMGO incubation at 1 and 100 nM (50.30 ± 9.71%, *p* = 0.0001 and 55.56 ± 24.20% of FK levels, *p* = 0.0005, respectively). DAMGO did not present dose-dependent effect either. Very surprisingly, naloxone, a specific, but non-selective OR antagonist did also significantly decrease FK-induced cAMP elevation (68.42 ± 6.87% of FK levels, *p* = 0.0394) when incubated alone at 1 µM with Caco-2 cells.

To confirm that the effect of hemorphins on cAMP production involves OR, we treated FK-stimulated cells with each hemorphin at 500 µM separately with or without naloxone at 1 µM. Unexpectedly, the naloxone treatment, when concomitant to hemorphins incubation, promoted a dramatically significant increase in FK-induced cAMP elevation (Figure 7B). It potentiated the FK effect on cAMP formation by at least 50% for each tested hemorphin as well as for DAMGO, and 38% for LVV-H4. Sidak *post hoc* analysis of each hemorphin or DAMGO treatment compared to its corresponding hemorphin or DAMGO and naloxone co-treatment yielded a statistically significant increase of cAMP of 93% for VV-H4 (*p* = 0.0006), 74% for VV-H7 (*p* = 0.0058), 93% for H7 (*p* = 0.0006), and 108% for DAMGO (*p* = 0.0184).

### Gene Expression Analysis (Caco-2 Model of IB)

**Figure 8:**
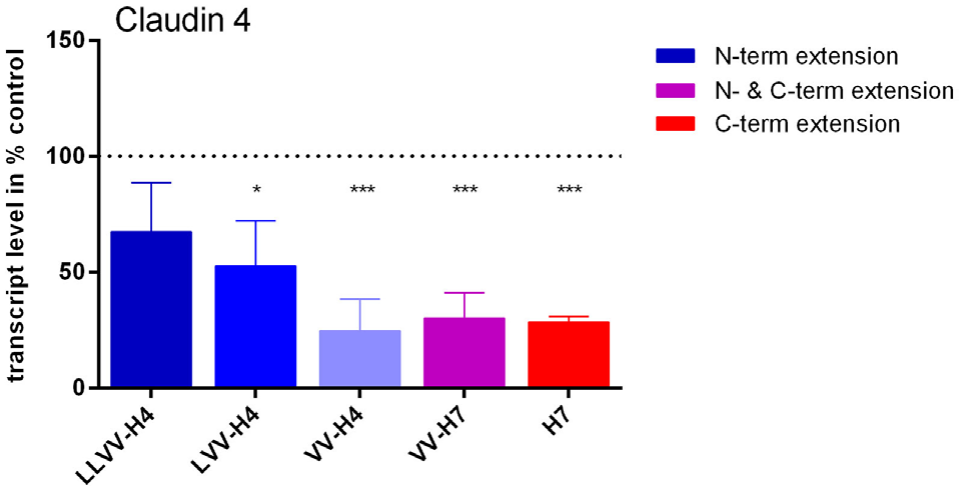
Effect of the five hemorphins on claudin-4 mRNA levels in the Caco-2 cells intestinal barrier (IB) model. Caco-2 cells were seeded on the filter of cell culture insert and cultured for 3 weeks until full differentiation as in the IB model. They were then incubated with 100 µM of each hemorphin/opioid peptide in culture medium for 24 h. Total RNAs were isolated and processed for quantitative real-time PCR analyses. Claudin-4 transcript levels were normalized by reference gene peptidylprolyl isomerase transcript levels and expressed as % of control levels (without opioid peptides). Experiments were run in triplicate and data represented are mean ± SD. *Statistically different from control; *p < 0.05, ***p < 0.005.

The potential effect of the five hemorphins on the TJ protein claudin-4 gene expression has been investigated *in vitro* on Caco-2 cells first grown for 3 weeks on cell culture membranes for full differentiation as epithelium. Quantitative PCR analyses showed that all hemorphins except LVV-H4 induced a significant decrease in claudin-4 mRNA levels of almost 50% or more of control levels (Figure 8). This effect was more pronounced in cells treated with hemorphins presenting a C-terminal extension than those harboring an N-terminal extension. The hemorphins presenting a C-terminal extension, VV-H7, and H7, reduced mRNA levels to 30.03 ± 10.99% (*p* = 0.0009) and 28.31 ± 2.52% (*p* = 0.0008) of control levels, respectively, VV-H4 to a similar extent with 24.74 ± 13.67% (*p* = 0.0005) and LVV-H4 to a lesser extent with 52.74 ± 19.37% (*p* = 0.0166) of control levels.

## Discussion

This study is a first report of the passage of food-derived hemorphins, i.e., exogenous hemorphins, through the intestinal as well as the BBB *in vitro* (Table 1). Information on the *in vitro* absorption of alimentary proteins or of well-defined peptide sequences derived thereof has been mainly garnered from tests with milk proteins (34–36), much less from tests with whey proteins (19, 37), or lately from egg albumin (38) but this information was unknown for hemorphins. The tested peptide the closest in terms of structure to hemorphins, is endomorphine-1 for which a low permeability through a caco-2 cell monolayer was reported (39).

We found that all hemorphins except the LLVV-H4 sequence could cross intact the Caco-2 cell monolayer. A possible explanation for the failure of passage of this sequence is brush-border enzymes degradation. The presence of multiple fragments in both apical and basal compartments may suggest that the peptides interacted with brush-border, basolateral, or intracellular peptidases (Figure 2). Indeed, LVV-H4, VV-H4, and H4 fragments and no LLVV-H4 detection in the apical compartment at the end of the experiment could reflect that LLVV-H4 was hydrolyzed to such an extent that no passage to the basal compartment occurred. It is recognized that the primary factor limiting transepithelial transport of intact peptides and its bioavailability is the susceptibility to cellular peptidases (40). This can reflect that the transport is probably concentration-dependent, since this was not the case in the LVV-H4 wells, where the fragment VV-H4 was detected both in apical and basal compartments. This does not exclude that this latter fragment is produced from LVV-H4 in apical and basal. However, the Caco-2 model is acknowledged as quite a stringent test for *in vitro* IB passage, meaning that results obtained in this test rather underestimate *in vivo* human IB passage (41).

Regarding the passage of the BBB, though applied at a much lower concentration than in the IB/Caco-2 test (100 µM vs 5 mM), all tested hemorphins proved to be able to cross intact the modeled BBB *in vitro*. Concentrations tested in each model were chosen according to a literature search among Caco-2 studies of compounds permeability. It yielded a range of concentrations tested between few hundreds of μM to few mM (100–500 × 10^−4^ to 10 × 10^−3^ M). A first trial concentration of 300 μM in the IB passage test resulted in no detectable recovery of the intact peptides in the basal compartment (data not shown). Given that if the hemorphins do cross the IB, they do so in very low amounts, therefore, it was physiologically relevant to test much lower concentrations in the BBB passage test. Moreover, in the BBB passage test, all intact hemorphins were detected in the basal compartment 30 min after their apical application in comparison to 60 min in the Caco-2 model, except for H7 which was detected already at 5 min in this latter model. In agreement with our results, the BBB passage of H7 had been demonstrated in a study using ^125^I-H7 in rats within 15 min of intra-femoral artery injection (42). However, a study comparing 18 peptides permeability through the BBB in rats in relation to their amino-acid structure and different physico-chemical properties (43) reported that N-terminal tyrosine constitutes a brake to BBB passage, yet H7 which presents such residue was clearly able to cross the *in vitro* BBB model in this study.

In addition, we show that fragments of the tested hemorphins were also able to pass the Caco-2 epithelium as well as the modeled BBB (Table 1) and that is interesting inasmuch as these fragments retain a certain bioactivity, like the OP-binding capacity. Strikingly, the most frequent hemorphin fragment recovered is the core tetrapeptide YPWT (H4), common to all tested hemorphins. Three pieces of evidence would indicate that YPWT is produced in the apical compartment by hydrolysis of the hemorphins and is able to cross the cell barrier. First, the fragment YPWT was identified in the basal samples as soon as 5 min incubation either before the parent peptide was even detected (LVV- and VV-YPWT) or at the same time (VV-YPWT-QRF and YPWT-QRF). Second, for LLVV-H4 wells, the parent peptide was seemingly not able to cross the cell barrier and for VV-H7, only traces were detected basally. Finally, 120 min apical amounts of YPWT were notably lower than basal ones. H4 was the first hemoglobin-derived opioid peptide identified as such (8) and also has the feature to be very stable in plasma.

In this regard, we verified the OR-binding capacity of the hemorphins synthesized for this study on rat brain membrane preparation. Our results add the sequence LVV-H4 as OR ligand, which, to the best of our knowledge, had not been tested for its opioid activity. Also, our results are in agreement with the findings of Zhao et al. (32) showing that N-extensions on the H4 core tend to disfavor OP-binding, whereas C-extensions tend to increase it.

We further sought to characterize the agonist/antagonist nature of the hemorphins in the cAMP assay commonly used for OR-ligand investigation. Multiple studies realized mostly with mu-OR agonists in different cell lines found that OR agonism resulted in a decrease of intracellular cAMP formation (33, 39, 44). Here, however, unexpected results were found with the naloxone treatment which is a well-known non-selective OR antagonist. Naloxone alone yielded decreased cAMP levels in FK-stimulated Caco-2 cells and potentiated cAMP increase when applied with each hemorphin (Figure 7). A comparable result was reported with naloxone in a different cell line as Fukuda et al. (45) showed that naloxone can have a partial agonist activity in mu- and kappa-transfected CHO-cells. Also, H4 was found as agonist in the GPI bioassay, but antagonist when applied in the ileum from a morphine-tolerant rat (i.e., after a morphine pre-treatment) (11). Hence, our results regarding the effect of hemorphins on the cAMP pathway in Caco-2 cells are compatible with an interaction with OR, but may also be indicative of the involvement of other G-protein-coupled receptors. Considering that hemorphins and naloxone effects separately inhibited FK-induced cAMP elevation, these results suggest a synergistic effect of hemorphins and naloxone on the cAMP pathway. Although performance of the cAMP assay in Caco-2 cells is not classical, this choice was justified by the use of these cells in the IB model and would thus allow us to infer some evidence regarding the underlying mechanisms of the hemorphins effects on TJs.

Indeed, hemorphins were suspected of passing the IB epithelium by interaction with the TJ. There is some evidence that some substances are able to alter intestinal permeability *via* their action on TJ proteins (40, 46, 47). TJ may be regulated by phosphorylation that promotes or decreases permeability. For instance, in low resistance cells the TJ protein ZO-1 is significantly more phosphorylated than in high resistance monolayers (48). Also, it has been shown that the phosphorylation of claudin-4 enhanced paracellular permeability in HT29 colon cells (49). Hence, our results support the potential action of hemorphins *via* OR and the cAMP pathway to modulate claudin and other TJ proteins at least in intestinal epithelial cells.

However, hemorphins could also have a long-term effect on TJ proteins that is related to the implication of claudins in intestinal pathologies when their expression or localization is altered (50). We show here that concentrations as low as 100 µM of each hemorphin could decrease the gene expression of claudin-4 in intestinal Caco-2 cells after 24 h of contact (Figure 8).

The digestion of dietary proteins produces peptides that have biological activities, here hemorphins that present OP-binding capacity [see also their intestinal luminal effects in Domenger et al. (16)]. These biopeptides, that resist digestion, also proved to be able to cross the intestinal and BBBs in *in vitro* conditions, intact, and as fragments that retain opioid-binding capacity. As such they could interact with peripheral and central OPs and participate in appetite and food intake regulation. Further studies will also specify the mechanisms of passage of these hemorphins, especially their interaction with TJ proteins and their impact on barrier permeability.

## Author Contributions

DD participated in all steps of preparation, realization, analyses, and writing of this manuscript. MK performed the LC-MS/MS analyses, MK and J-FG provided guidance in the interpretation of the LC-MS/MS results. VT contributed to the passage tests in the Caco-2 model realization. CL contributed to the passage tests in the BLEC model realization. SL, CL, and FG helped in the critical review and the editing of the manuscript. JL helped in the RIA experiments and in the editing of the manuscript. PD helped in the editing of the manuscript. BC and RR were awarded the funding and helped in the design of the experiments and editing the manuscript.

## Conflict of Interest Statement

The authors declare that the research was conducted in the absence of any commercial or financial relationships that could be construed as a potential conflict of interest.
